# Superiority of Laparoscopic Gastrojejunostomy Combined With Multimodality Therapy for Gastric Outlet Obstruction Caused by Advanced Gastric Cancer

**DOI:** 10.3389/fonc.2022.814283

**Published:** 2022-01-28

**Authors:** Chuandong Wang, Xiaojuan Zhang, Shengtao Lin, Changshun Yang, BiaoHuan Zhou, Yulong Mi, Rong Ye, Yifan Chen, Weijie Chen, Xiaojun Lin, Song Tan, Yuhang Zhou, Weihua Li

**Affiliations:** ^1^Shengli Clinical Medical College of Fujian Medical University, Fuzhou, China; ^2^Department of Surgical Oncology, Fujian Provincial Hospital, Fuzhou, China; ^3^Fuzong Clinical Medical College of Fujian Medical University, Fuzhou, China; ^4^Department of Radiology, 900th Hospital Logistic Support Forces of PLA, Fuzhou, China

**Keywords:** gastric cancer, gastrojejunostomy, endoscopy, neoadjuvant chemotherapy, conversion therapy

## Abstract

**Background:**

Data are limited concerning the survival outcomes of patients with gastric outlet obstruction (GOO) caused by advanced gastric cancers according to laparoscopic gastrojejunostomy (LGJ) combined with multimodality therapy (MMT). Therefore, the purpose of this study was to examine the feasibility and efficacy of these therapies.

**Methods:**

This single-centered, retrospective analysis included data of 184 patients with GOO due to advanced gastric cancer (AGC). Treatment models were: laparoscopic gastrojejunostomy combined with multimodality therapy (LGJ+MMT), endoscopic metal stent placement combined with multimodality therapy (EMSP+MMT), and multimodality therapy (MMT).

**Results:**

Improved oral intake, better nutritional indices, and better response to chemotherapy were observed in the LGJ+MMT group. Subsequent gastrectomy was performed in 43 (61.4%) patients in the LGJ+MMT group, 23 (37.7%) in the EMSP+MMT group, and 11 (20.8%) in the MMT group (P<0.001). LGJ+MMT was associated with better long-term prognosis. As confirmed by propensity scores and multivariate analyses, the 3-year survival rates in the three treatment models were 31.4% with LGJ+MMT, 0% with EMSP+MMT, and 0% with MMT in conversion therapy, and 50.0% with LGJ+MMT, 33.3% with EMSP+MMT, and 23.5% with MMT in NAC. A forest plot revealed that LGJ+MMT was related to a decreased risk of death.

**Conclusions:**

LGJ combined with MMT was associated with better nutritional status, higher rates of subsequent gastrectomy, and good prognosis. LGJ combined with MMT may improve the long-term survival of patients with GOO caused by AGC.

## Introduction

Gastric cancer is frequently diagnosed at an advanced stage with a poor prognosis (1). Multimodality therapy (MMT), which is defined as neoadjuvant chemotherapy (NAC) therapy or conversion therapy, is a therapeutic regimen for advanced gastric cancer (AGC) (2–4).

Several landmark clinical trials have revealed the survival benefits of MMT for advanced gastric cancer. The MAGIC trial showed an improved 5-year survival rate (23% to 36%) for advanced gastric cancer treated with perioperative chemotherapy, revealing the era of neoadjuvant chemotherapy (5). In addition, some patients with initially unresectable tumors who responded to palliative chemotherapy underwent conversion surgery in the REGATTA trial, with a better long-term outcome (6). Similar findings have been reported in numerous investigations, and each of these trials demonstrated prolonged survival of AGC treated with MMT (2, 4, 7–12). However, gastric outlet obstruction (GOO) is a common and detrimental complication of AGC (3, 13), which deprives patients the opportunity to undergo MMT with deteriorated nutritional and metabolic patterns (3). To address this issue, alleviating GOO plays a vital role in the application of MMT. Laparoscopic gastrojejunostomy (LGJ) is a promising option in restoring oral intake with small incisions, reduced immunosuppression, and enhanced compliance with chemotherapies (13, 14). Our institution recently published two studies on MMT with LGJ followed by conversion therapy and demonstrated higher conversion surgery completion rates in patients with GOO caused by incurable AGC (48.6% and 47.9%) (15, 16). However, little is known regarding treatment models with LGJ followed by neoadjuvant therapy in patients with GOO due to AGC. Moreover, there remains no head-to-head comparison of LGJ and endoscopic stenting in patients with GOO receiving MMT.

Therefore, the purpose of the present research was to compare MMT completion rates and prognosis in these treatment models and identify factors associated with survival to verify the feasibility and efficacy of these treatments.

## Method

### Patient Selection

We retrospectively reviewed clinical data of patients with GOO due to AGC in Fujian Provincial Hospital between June 2015 and June 2020. We retrieved data of eligible patients for analysis based on the following criteria: histologic and radiologic confirmation of AGC; endoscopic confirmation of GOO with difficulty in oral intake; at least two cycles of chemotherapy before gastrectomy; 20–80 years of age; good tolerance of general anesthesia; Eastern Cooperative Oncology Group performance status (PS) score of 0–2; and no prior chemotherapy, targeted therapy, or radiotherapy. We included patients with initial unresectable and locally advanced gastric cancer who had indications for NAC and conversion therapy. Patients with early stage (T1N0) disease, gastric cancer perforation, active bleeding, combined with other malignant tumors, altered chemotherapy regimen, and incomplete data were excluded. This study was reviewed and approved by the Ethics Committee of Fujian Provincial Hospital. Data were anonymized, and the requirement for informed consent from the patients was waived. All study procedures were performed in accordance with the Helsinki Declaration of 1964 and later versions.

### Procedural Details

A multidisciplinary team consisting of oncology, nutrition, and surgery experts determined the strategy for each patient. Patients with a GOOSS score of 2 were categorized into the MMT group, while patients with a GOOSS score of 0 or 1 were categorized into LGJ+MMT or EMSP+MMT. After LGJ or EMSP, enteral nutrition and early parenteral nutrition was initiated. All patients received additional enteral nutrition support during the hospitalization. On postoperative day 1, patients were encouraged to drink 500–1000 ml of clear fluid. The amount of fluid intake was increased as tolerated by patients. Parenteral nutrition was discontinued when oral intake reached 2000–2500 ml/day. All patients received nutrition (protein 4.0 g, fat 3.0 g, carbohydrate 12.1 g, caloric value 1.0 kcal/ml) at a temperature of 40°C in the hospital. The calorie and protein intake were 25–30 kcal/kg/day and 1–2 g/kg/day, respectively, supplemented by parenteral nutrition (15, 16). EOX therapy was applied 7–14 days after LGJ or EMSP, which consisted of oxaliplatin 130 mg/m^2^ intravenously on day 1 and epirubicin 100 mg/m^2^ intravenously on day 1, with fluoropyrimidine capecitabine 825 mg/m^2^ orally twice daily on days 1–14. Preoperative chemotherapy was generally continued for 2–4 cycles in patients treated with NAC and 6–8 cycles in patients treated with conversion therapy. Tumor response was evaluated using abdominal enhanced computed tomography every two cycles of chemotherapy. Treatment was discontinued in cases of tumor progression, patient refusal, and unacceptable chemotherapy toxicity. The multidisciplinary team determines the criteria for gastrectomy when CR or PR is generally observed (10). Adjuvant chemotherapy was determined by the attending physicians in a clinical setting.

### Data Collection

Patient information and clinical and pathological characteristics were obtained from the electronic medical records. Demographic and preoperative variables were acquired, including age, sex, performance status (PS), body mass index (BMI), nutritional and inflammatory status, clinical stages, and GOOSS. GOOSS is defined as follows: 0, no oral intake; 1, liquid only; 2, soft food; and 3, low-residue or full diet (14). Nutritional status was estimated using Onodera’s prognostic nutritional index (PNI) and BMI. Inflammatory status was estimated by the neutrophil-to-lymphocyte ratio (NLR) and platelet-to-lymphocyte ratio (PLR). According to previous studies, we divided patients into two groups based on PNI (<45 or ≥45), PLR (<162 or ≥162), and NLR (<2.5 or ≥2.5) (17–21). Clinical and pathological stages were determined according to the National Comprehensive Cancer Network (NCCN) guidelines (22). Response to chemotherapy was classified according to the Response Evaluation Criteria in Solid Tumors guidelines (version 1.0) (12).

### Statistical Analysis

Statistical analyses were conducted using SPSS software (IBM SPSS Statistics for Windows, version 22.0, Armonk, NY, USA). Two-tailed *P* values <0.05 were considered significant. Wilcoxon rank-sum tests were used for continuous variables and the Pearson chi-square test was used for categorical variables. Prognostic factors of overall survival (OS) were analyzed using univariate and multivariate logistic regression. OS rates were calculated using the Kaplan–Meier method. A propensity score-matched analysis was conducted to avoid confounding bias (performance status) with a small caliper of 0.2. Subgroup analyses were used to evaluate the impact of treatment models on OS after LGJ+MMT vs. EMSP+MMT.

## Result

### Baseline Characteristics

During the study period, we identified 224 patients with GOO caused by AGC. Forty patients were excluded due to an altered chemotherapy regimen (n=13), less than two cycles of chemotherapy (n=11), missing data (n=8), and other treatments (n=8). We obtained data on 70 patients who received LGJ+MMT, 61 patients who received EMSP+MMT, and 53 patients who received MMT only (**Table 1**). The EMSP+MMT group had a lower performance status (*P*<0.001). More than three-quarters (82.0%) of the EMSP+MMT group had a preoperative PS of 2, in contrast to only 37.1% in the LGJ+MMT and 54.7% in the MMT group. Significant differences were not found in ratios of PNI≥45, which represent nutritional status, and the ratios of PLR <162 and NLR <2.5, which represent the inflammatory status, in these treatment models (17–23). There was also no significant difference in the distribution of tumor stages, non-curable factors, and MMT regimens.

**Table 1:** Baseline Patients Characteristics.

	LGJ+MMT (n=70)	EMSP+MMT (n=61)	MMT (n=53)	*P value*
Age (year)	62 (33-80)	67 (32-80)	57 (28-77)	0.073
Sex (male/female)	48/22 (68.6/31.4)	46/15 (75.4/24.6)	35/18 (66.0/44.0)	0.518
PS (0/1/2)	8/36/26 (11.4/51.4/37.1)	3/8/50 (4.9/13.1/82.0)	4/20/29 (7.5/37.7/54.7)	<0.001
BMI	21.3 (17.3-26.4)	21.6 (17.7-27.8)	22.5 (18.2-29.0)	0.078
GOOSS (0/1/2)	29/41/0 (41.4/58.6/0)	39/22/0 (63.9/36.1/0)	0/0/53 (0/0/100)	<0.001
PNI				0.970
<45	51 (72.9)	45 (73.8)	38 (71.7)	
≥45	19 (27.1)	16 (26.2)	15 (28.3)	
PLR				0.413
<162	21 (30.0)	21 (34.4)	22 (41.5)	
≥162	49 (70.0)	40 (65.6)	31 (58.5)	
NLR				0.144
<2.5	19 (27.1)	16 (26.2)	22 (41.5)	
≥2.5	51 (72.9)	45 (73.8)	31 (58.5)	
cT				0.928
T2	1 (1.4)	1 (1.6)	1 (1.9)	
T3	10 (14.3)	12 (19.7)	7 (13.2)	
T4a	51 (72.9)	41 (67.2)	41 (77.4)	
T4b	8 (11.4)	7 (11.5)	4 (7.5)	
cN (+)	70 (100)	61 (100)	53 (100)	–
Non-curable factor				
Infiltration to adjacent organs	3 (4.3)	5 (8.2)	4 (7.5)	0.623
Peritoneal metastasis	34 (48.6)	24 (39.3)	19 (35.8)	0.326
Hepatic metastasis	10 (14.3)	8 (13.1)	9 (17.0)	0.839
Distant lymph node metastasis	28 (40.0)	23 (37.7)	23 (43.4)	0.825
MMT (NAC/Conversion)	18/52 (25.7/74.3)	12/49 (19.7/80.3)	17/36 (32.1/67.9)	0.317

PS, Performance status; GOOSS, Gastric outlet obstruction scoring system; PNI, Prognostic Nutritional Index; BMI, Body mass index; PLR, Platelet to lymphocyte ratio; NLR, Neutrophil to lymphocyte ratio; MMT, multimodality therapy.

### Clinical and Pathologic Outcomes

**Table 2** summarizes the clinical outcomes of each treatment model, which were collected after two cycles of chemotherapy. Significant improvements in oral intake were observed after treatment with LGJ and EMSP. GOOSS 3 was achieved in 98.6% of the LGJ+MMT group and 86.9% of the EMSP+MMT group after the intervention. However, none of the patients in the MMT group had a restored full diet. Patients treated with LGJ+MMT received more cycles of chemotherapy, especially in conversion therapy (six cycles vs. two cycles vs. three cycles, *P*<0.001). In addition, the proportion of patients with PNI ≥45 was significantly higher in the LGJ+MMT group than in the other groups (64.3% vs. 54.1% vs. 35.8%, *P*=0.007). These results were attributed to 61.4% of the LGJ+MMT group who displayed a major response (5.7% complete response and 55.7% partial response). Notably, no significant differences were found in chemotherapy cycles of patients treated with NAC. In contrast, higher rates of PLR <162 and NLR <2.5 were observed in the MMT group (34.3% vs. 19.7% vs. 60.4%, and 55.7% vs. 34.4% vs. 73.6%, respectively, *P<0.001*).

**Table 2:** Clinical outcomes after treatment models.

	LGJ+MMT (n=70)	EMSP+MMT (n=61)	MMT (n=53)	*P* value
GOOSS 3 achieved	69 (98.6)	53 (86.9)	0 (0)	<0.001
Chemotherapy cycles				
NAC	4 (2-4)	4 (2-4)	4 (1-4)	0.251
Conversion	6 (2-10)	2 (2-8)	3 (2-6)	<0.001
BMI	21.2 (17.4-27.7)	21.6 (17.7-27.8)	22.2 (18.0-27.8)	0.322
PNI				0.007
<45	25 (35.7)	28 (45.9)	34 (64.2)	
≥45	45 (64.3)	33 (54.1)	19 (35.8)	
PLR				<0.001
<162	24 (34.3)	12 (19.7)	32 (60.4)	
≥162	46 (65.7)	49 (80.3)	21 (39.6)	
NLR				<0.001
<2.5	39 (55.7)	21 (34.4)	39 (73.6)	
≥2.5	31 (44.3)	40 (65.6)	14 (26.4)	
Response				
Complete response	4 (5.7)	2 (3.3)	0 (0)	
Partial response	39 (55.7)	21 (34.4)	11 (20.8)	
Stable disease	5 (7.1)	21 (34.4)	25 (47.2)	
Progressive disease	22 (31.4)	17 (27.9)	17 (32.0)	
ORR (%)	61.4	37.7	20.8	<0.001
Subsequent resection	43 (61.4)	23 (37.7)	11 (20.8)	<0.001

GOOSS, Gastric outlet obstruction scoring system; NAC, Neoadjuvant chemotherapy; PNI, Prognostic Nutritional Index; BMI, Body mass index; PLR, Platelet to lymphocyte ratio; NLR, Neutrophil to lymphocyte ratio; MMT, multimodality therapy; ORR, Objective response rate.

Subsequent gastrectomy was performed in 43 (61.4%) patients in the LGJ+MMT group, 23 (37.7%) in the EMSP+MMT group, and 11 (20.8%) in the MMT group. There were 12 and six cases of peritoneal metastasis in the LGJ+MMT and EMSP+MMT groups, respectively, which disappeared after chemotherapy. In the LGJ+MMT group, three and four patients with hepatic metastasis underwent additional radiofrequency ablation and combined partial hepatectomy, respectively. Additional radiofrequency ablation and combined partial hepatectomy were performed in one and three cases in the EMSP+MMT group, respectively. Among patients with organ infiltrations, one case of infiltration lesion disappeared and two patients underwent partial pancreatectomy in the LGJ+MMT group. In contrast, two patients in the EMSP+MMT group underwent partial pancreatectomy. None of the patients with non-curable factors treated with conversion therapy underwent subsequent resection in the MMT group. In patients treated with NAC, 17 (94.4%), 9 (75.0%), and 11 (64.7%) patients received subsequent gastrectomy in the LGJ+MMT, EMSP+MMT, and MMT groups, respectively. No significant differences were found in the pathological outcomes of the treatment models (**Table 3**).

**Table 3:** Surgical and pathological findings after treatments models.

	LGJ+MMT (n=43)	EMSP+MMT (n=23)	MMT (n=11)	*P* value
Resection margin				0.945
R0	38 (88.4)	20 (87.0)	10 (90.9)	
R1	5 (11.6)	3 (13.0)	1 (9.1)	
Pathological response				0.204
0	4 (9.3)	3 (13.0)	0 (0)	
1	18 (41.9)	13 (56.6)	4 (36.4)	
2	10 (23.3)	4 (17.4)	6 (54.5)	
3	11 (25.6)	3 (13.0)	1 (9.1)	
pT				0.147
T0	4 (9.3)	1 (4.3)	0 (0)	
T2	1 (2.3)	3 (13.0)	3 (27.3)	
T3	8 (18.6)	6 (26.1)	1 (9.1)	
T4a	30 (69.8)	13 (56.6)	7 (63.6)	
pN				0.109
N0	25 (58.2)	9 (39.2)	6 (54.5)	
N1	8 (18.6)	11 (47.8)	1 (9.1)	
N2	5 (11.6)	2 (8.7)	3 (27.3)	
N3	5 (11.6)	1 (4.3)	1 (9.1)	

### Survival Analysis

**Figure 1:**
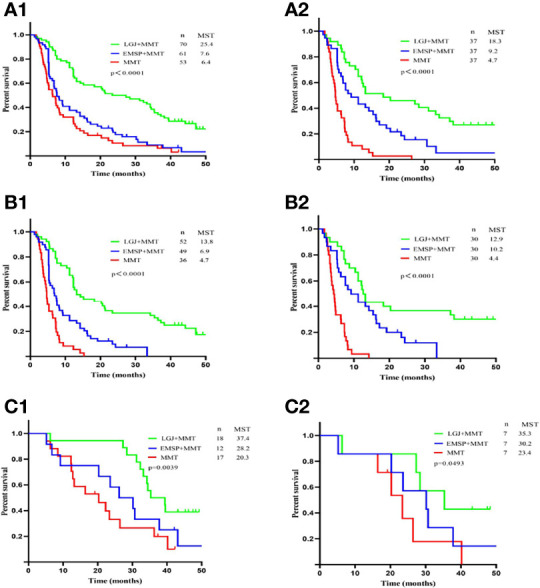
Overall survival according to treatment models. **(A1)** OS for patients treated by NAC and conversion therapy. **(A2)** OS for patients treated by NAC and conversion therapy after propensity score matching. **(B1)** OS for patients treated by conversion therapy. **(B2)** OS for patients treated by conversion therapy after propensity score matching. **(C1)** OS for patients treated by NAC therapy. **(C2)** OS for patients treated by NAC therapy after propensity score matching.

**Figure 2:**
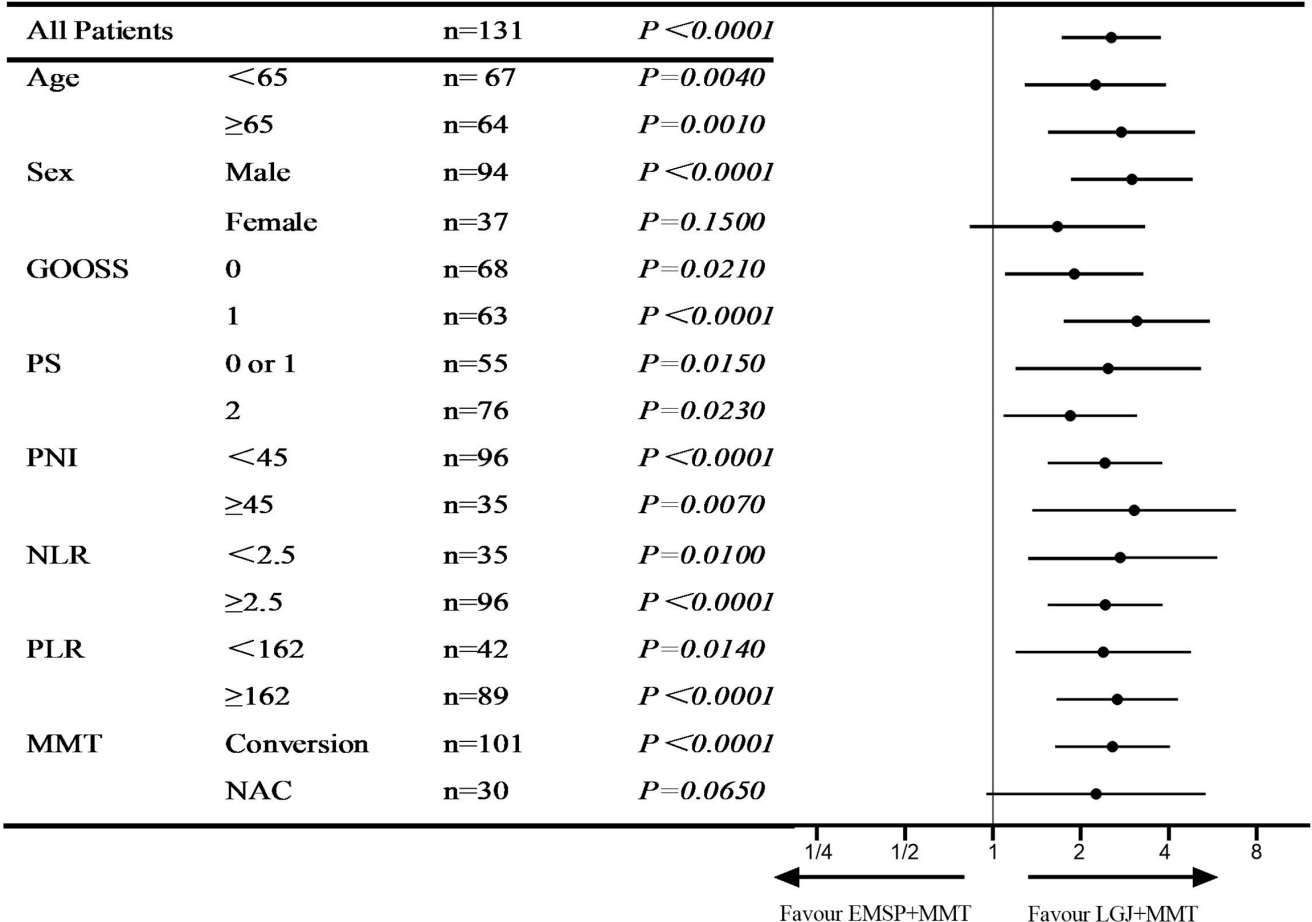
Hazard ratio for overall survival. Forest plot evaluating the impact of treatment models on OS after LGJ+MMT vs EMSP+MMT.

We compared the OS between the three groups, and significant differences in median survival time (MST) were found in patients treated with NAC (37.4 vs. 28.2 vs. 20.3 months, *P*=0.0039), conversion therapy (13.8 vs. 6.9 vs. 4.7 months, *P* < 0.0001), and both treatments (25.4 vs. 7.6 vs. 6.4 months, *P*<0.0001). We performed propensity score matching to reduce the selection bias (**Figure 1**). Outcomes data after matching also demonstrated that the LGJ+MMT group had a better prognosis (*P*<0.05). In addition, the 3-year survival rates were noteworthy across the three treatment models: 31.4% with LGJ+MMT, 0% with EMSP+MMT, and 0% with MMT in conversion therapy, and 50.0% with LGJ+MMT, 33.3% with EMSP+MMT, and 23.5% with MMT in NAC. Regardless of treatment models, patients who completed subsequent gastrectomy had improved OS compared to those who did not finish the treatment (MST: 32.8 vs. 6.5 months, *P*<0.001). This was confirmed again in the univariate and multivariate analyses (HR, 48.783: 95% CI: 19.546–121.754, *P*<0.001). Compared with LGJ+MMT, EMSP+MMT (HR, 2.242; 95% CI: 1.460–3.441, *P*<0.001) and MMT (HR, 2.199; 95% CI: 1.395–3.468, *P*=0.001) were associated with an increased risk of death. An additional factor that increased the risk of death was conversion therapy (HR, 1.589; 95% CI 1.030–2.452, *P*=0.036) (**Table 4**). A forest plot revealed that LGJ+MMT was related to a decreased risk of death in all subgroups (**Figure 2**).

**Table 4:** Univariate and multivariate analyses for OS.

Variable	Hazard ratio	95% CI	*P* value
Univariate analysis			
Age (≥65/<65)	0.837	0.614-1.141	0.260
Sex (male/female)	1.283	0.917-1.795	0.145
PS (2/0 or 1)	0.490	0.356-0.676	<0.001
BMI (≥18.5/<18.5)	1.340	0.705-2.545	0.372
PNI (≥45/<45)	1.008	0.710-1.431	0.967
PLR (≥162/<162)	1.166	0.843-1.613	0.353
NLR (≥2.5/<2.5)	0.891	0.639-1.243	0.396
Subsequent resection (yes/no)	67.736	27.536-166.623	<0.001
MMT (NAC/conversion)	2.366	1.623-3.450	<0.001
Treatment selection			
LGJ+MMT	Ref	Ref	Ref
EMSP+MMT	2.424	1.646-3.569	<0.001
MMT	3.195	2.135-4.780	<0.001
Multivariate analysis			
PS (2/0 or 1)	0.775	0.542-1.108	0.163
Subsequent resection (yes/no)	48.783	19.546-121.754	<0.001
MMT (NAC/conversion)	1.589	1.030-2.452	0.036
Treatment selection			
LGJ+MMT	Ref	Ref	Ref
EMSP+MMT	2.242	1.460-3.441	<0.001
MMT	2.199	1.395-3.468	0.001

PS, Performance status; PNI, Prognostic Nutritional Index; BMI, Body mass index; PLR, Platelet to lymphocyte ratio; NLR, Neutrophil to lymphocyte ratio; MMT, multimodality therapy.

## Discussion

The survival benefits of MMT have been demonstrated in numerous large, multicentered, prospective trials of AGC (5, 6). Significantly improved survival of more than 40 months could be achieved in patients with incurable AGC treated with conversion therapy (4). However, the major challenge in these trials was the ability of patients to receive the intended MMT (2). GOO, a common complication of distal AGC, impairs the ability to receive MMT (13). We previously showed that LGJ combined with conversion therapy is an independent prognostic factor of OS in patients with GOO caused by incurable AGC. However, whether such treatment could prolong survival in all patients with GOO due to AGC has not been clarified. To the best of our knowledge, the present study is the first to compare the long-term prognosis of different methods to alleviate GOO immediately before MMT and evaluate the effect of these treatment models on OS. Our study revealed a marked improvement in eating practices and better nutritional status, and response to chemotherapy after LGJ, similar to findings of previous studies (15, 16). Furthermore, the MST of the LGJ+MMT group was also higher than that of the EMSP+MMT and MMT groups for both NAC and conversion therapy. Multivariate analysis identified that EMSP+MMT and MMT were associated with an increased risk of death, compared with LGJ+MMT. These findings suggest that LGJ combined with MMT could achieve better long-term survival in these patients.

Previous studies on GOO made more efforts on short-term outcomes, including restoration of oral intake, postoperative complications, and luminal patency duration. The reported advantages of EMSP include rapid resumption of oral intake and shorter hospital stay, which are more likely to be used in patients with physical deterioration. Several studies have also demonstrated longer luminal patency durations and lower intervention rates after surgery (3, 13, 24). In addition, with the advancement of laparoscopy, the median time of resumption of oral intake reduces to 2 and 4 days, as reported by some studies (25–27). However, these studies, under consideration for palliative purposes, did not address long-term survival. Furthermore, many investigators have revealed that completion of MMT, especially curative surgery, plays a vital role in the long-term prognosis of AGC (2, 4–12). Yoshio et al. (3) conducted a multicentered cohort study on patients with GOO receiving stents and gastrojejunostomy, and found that only 1% and 15% of patients underwent subsequent resection, respectively. In addition, Tanaka et al. (14) demonstrated that only 13.3% of patients with GOO underwent conversion surgery after LGJ. This may suggest that long-term malnutrition caused by GOO impairs the ability of patients to undertake subsequent treatments, especially in conversion therapy, which requires more cycles of chemotherapy. The true advantages of these interventions hinge on the restoration of nutritional and metabolic status. In this current study, LGJ+MMT have significantly improved subsequent resection rates compared with EMSP+MMT and MMT (61.4% vs. 37.7% vs. 20.8%, *P<0.0001*), in addition to higher rates of PNI ≥45 (64.3% vs. 54.1% with EMSP+MMT and 35.8% with MMT), lower rates of PLR <162 (34.3% vs. 19.7% with EMSP+MMT), and lower rates of NLR <2.5% (55.7% vs. 34.4% with EMSP+MMT). This phenomenon may be related to the immediate application of enteral nutrition after LGJ or EMSP combined with early parenteral nutrition. Interestingly, reduced inflammatory status was observed in the MMT group, which may result from myelosuppressive effects of cytotoxic anticancer chemotherapy, especially in patients with a lower nutritional status (28).

In survival analysis, LGJ+MMT offers a survival benefit over EMSP+MMT and MMT in patients with obstructive AGC, and propensity score matching strengthens this hypothesis (*P<0.05*). In addition, multivariate analysis identified that treatment with EMSP+MMT (HR, 2.242; 95% CI: 1.460–3.441, *P<*0.001) and MMT (HR, 2.199; 95% CI 1.395–3.468, *P*=0.001) were associated with an increased overall risk of death, compared with LGJ+MMT. Previous studies have demonstrated that gastrojejunostomy can enhance compliance with chemotherapy and is associated with better nutritional and metabolic status (13–16), arguing that the primary survival advantages of this treatment model were due to more cycles of chemotherapy. In our current study, significantly increased cycles of chemotherapy were observed in patients treated with conversion therapy after LGJ (six cycles vs. two cycles vs. three cycles, *P*<0.001). However, no significant differences were found in patients receiving NAC (*P=0.251*), which may be due to the shorter time these patients needed. However, our propensity score-matched study revealed long survival in patients treated with LGJ+MMT in NAC (35.3 vs. 30.2 vs. 23.4 months, *P*=0.0493). This phenomenon may be due to better nutrition and inflammatory status, represented by increased PNI and decreased NLR and PLR (17–21). In particular, the difference in pathological states after treatment was not found, which can be explained by the selection bias that subsequent resection involves only those patients who respond to chemotherapy and subsequently undergo surgery. The question then arises on the optimum personalized enteral nutrition after LGJ and suitably alters the chemotherapy regimen when chemotherapy fails.

One concern with methods to alleviate GOO lies in the indications to choose surgery or endoscopy. A previous study demonstrated that stent therapy was selected for more physically deteriorated patients who underwent gastrojejunostomy in clinical settings (3). However, this result was limited to open operation and was focused on palliative purposes. In this study, LGJ+MMT was associated with a better prognosis in patients with GOO. To further evaluate the impact of treatment models on the risk of death, we performed a subgroup analysis and found that patients who underwent LGJ+MMT had a decreased risk of death in any subgroup.

This study has several limitations. First, although propensity score matching was used to balance the significant baseline characteristics of the patients, RCTs are desirable for further analysis. Moreover, this study had a relatively small sample size and a retrospective exploratory design. Second, we excluded patients with less than two cycles of chemotherapy and altered chemotherapy regimens. Since the target patients had deteriorated nutritional and metabolic status, difficulties were associated with obtaining and maintaining subsequent treatments. Finally, the follow-up period was not long enough to achieve a 5-year survival rate.

## Conclusion

We demonstrated that in patients with GOO, LGJ+MMT improved nutritional and inflammatory status, increased subsequent resection rates, and at the 3-year of follow-up, has survival benefits compared to EMSP+MMT and MMT. In the absence of randomized controlled trials directly comparing these treatment models, we conclude that LGJ+MMT is a feasible and effective modality for treating GOO caused by AGC. Further investigations should be conducted develop personalized scheme to implement this strategy.

## Data Availability Statement

All data and materials generated during and/or analyzed during the current study are available from the corresponding author on reasonable request.

## Ethics Statement

The studies involving human participants were reviewed and approved by the Ethics Committee of Fujian Provincial Hospital. Written informed consent for participation was not required for this study in accordance with the national legislation and the institutional requirements.

## Author Contributions

Conceptualization, WL. Methodology, CW, XZ, and SL. Formal analysis, CW, XZ, CY, BZ, YM, RY, YC, WC, XL, ST, YZ, and WL. Resources, SL, CY, BZ, and WL. Writing—original draft preparation. CW, XZ, and WL. Writing—review and editing. CW, XZ, and WL. Funding acquisition, WL. All authors have read and agreed to the published version of the manuscript.

## Conflict of Interest

The authors declare that the research was conducted in the absence of any commercial or financial relationships that could be construed as a potential conflict of interest.

## Publisher’s Note

All claims expressed in this article are solely those of the authors and do not necessarily represent those of their affiliated organizations, or those of the publisher, the editors and the reviewers. Any product that may be evaluated in this article, or claim that may be made by its manufacturer, is not guaranteed or endorsed by the publisher.
